# PRDM14在非小细胞肺癌中的表达及其与临床病理因素的关系

**DOI:** 10.3779/j.issn.1009-3419.2010.09.06

**Published:** 2010-09-20

**Authors:** 冰冰 刘, 四洋 张, 林萍 惠, 雪杉 邱, 泽实 崔

**Affiliations:** 1 110001 沈阳，中国医科大学实验技术中心三部 The Center of Laboratory Technology and Experimental Medicine, Shenyang 110001, China; 2 110032 沈阳，中国医科大学附属第四医院中心实验室 The Center Laboratory of the Fourth Affliated Hospital of China Medical University, Shenyang 110032, China; 3 110001 沈阳，中国医科大学附属第一医院病理科 Department of Pathology, the First Affliated Hospital of China Medical University, Shenyang 110001, China

**Keywords:** 肺肿瘤, PRDM14, 组织学分型, 分化, Lung neoplasms, *PRDM14* gene, Histological type, Differentiation

## Abstract

**背景与目的:**

锌指蛋白转录抑制因子(positive regulatory domain zinc finger protein, PRDM)是一个有关人类肿瘤形成的转录调节因子家族，在细胞分化和恶性变中发挥重要作用。PRDM14是PRDM家族的成员之一。本研究的目的是检测PRDM14在非小细胞肺癌组织中的表达情况并探讨其与非小细胞肺癌的临床病理因素的关系。

**方法:**

采用免疫组织化学方法检测70例非小细胞肺癌标本和7例癌旁组织中PRDM14的表达。采用Western blot方法检测42例非小细胞肺癌组织和癌旁肺组织中PRDM14蛋白的表达。

**结果:**

7例癌旁肺组织中PRDM14弱表达，在70例非小细胞肺癌组织标本中，有8例为PRDM14阴性表达(11.43%)，9例为PRDM14弱阳性表达(12.86%)，36例PRDM14阳性表达(51.43%)，有17例PRDM14强阳性表达(24.29%)，PRDM14的表达情况与非小细胞肺癌的分化程度(*P*=0.046)和组织学类型(*P*=0.047)有关，PRDM14在高分化腺癌、鳞癌中表达最高，在中分化腺癌、鳞癌中表达次之，在低分化腺癌、鳞癌表达最低，PRDM14在腺癌中的表达高于鳞癌。Western blot结果表明PRDM14的蛋白在癌旁肺组织和肺腺癌、鳞癌组织的表达水平存在显著差异，PRDM14在非小细胞肺癌组织中的表达水平高于癌旁肺组织(*P*＜0.001)，而且与非小细胞肺癌的分化程度有关(*P*=0.017)，在高、中分化腺癌、鳞癌中的表达高于在低分化腺癌、鳞癌中的表达。

**结论:**

PRDM14在癌旁肺组织中低表达，在非小细胞肺癌组织中高表达，其表达水平与非小细胞肺癌的分化、组织学类型有关，可能在非小细胞肺癌的发生发展中发挥作用。

## Abstract (en)

**Background and objective:**

The positive regulatory domain proteins (PRDM) are family of transcriptional regulation related to the formation of human tumor factor and play key roles in the cell differentiation and malignant transformation. PRDM14 is a member of the PRDM family. The aim of this study is to detect the expression of PRDM14 in non-small cell lung cancer (NSCLC) tissues, and analyze its relationship with clinicopathologic characteristics of NSCLC.

**Methods:**

PRDM14 expression was detected in 70 NSCLC specimens and 7 paracancerous tissues using the immunohistochemistry (SP method). The PRDM14 protein expression was determined in 42 NSCLC specimens and 42 paracancerous tissues by Western blot.

**Results:**

Among 70 NSCLC specimens, 8 specimens showed weak expression of PRDM14 (11.43%, 8/70), 62 specimens showed moderate to strong staining of PRDM14 (88.57%, 62/70), whereas 7 paracancerous specimens showed weak staining extent. PRDM14 expression level was positively correlated with differentiation (*P*=0.046) and histological type (*P*=0.047). The positive cytoplasmic expression of PRDM14 in highly differentiated NSCLC, the low expression of PRDM14 in poorly differentiated NSCLC. The results of Western blot showed that there were significant difference between the two groups (*P* < 0.001); expression of PRDM14 was conspicuous in NSCLC specimens but low in paracancerous tissues. PRDM14 expression level was positively correlated with differentiation (*P*=0.017). The positive cytoplasmic expression of PRDM14 in highly differentiated NSCLC, the low expression of PRDM14 in poorly differentiated NSCLC.

**Conclusion:**

The high expression of PRDM14 in NSCLC is associated with differentiation and histological type. The PRDM14 may play an important role in the development of NSCLC.

锌指蛋白转录抑制因子(positive regulatory domain zinc finger protein, PRDM)是一个新确认的蛋白序列，是一个与人类肿瘤形成有关的转录调节因子家族^[1]^。尽管PRDM在转录调节中的作用还不十分清楚，但近年来有研究^[2]^表明PRDM家族的成员在细胞分化和恶性变中发挥重要作用。PRDM14是PRDM家族成员之一，具有1个PR-domain和6个锌指结构的蛋白。PRDM14位于染色体8q13.3上。最近有研究^[3]^通过基因芯片鉴定PRDM14在特定的未分化的人类胚胎干细胞中表达。Noriko等^[4]^研究发现在正常乳腺组织里几乎没有PRDM14的表达，而在乳腺癌中PRDM14常常过表达，异常表达的PRDM14促进了肿瘤细胞的生长并降低了肿瘤细胞对化疗药物的敏感性。而且据Dettman等^[5]^的研究，PRDM14在Evi32原病毒整合的肿瘤中过表达。但PRDM14在非小细胞肺癌中的表达情况尚不明了。本研究选择非小细胞肺癌患者为研究对象，检测PRDM14在不同组织学类型非小细胞肺癌和不同非小细胞肺癌细胞系中的表达情况。

## 1 材料和方法

### 1.1 材料

70例非小细胞肺癌组织石蜡标本及7例癌旁肺组织标本用于免疫组化检测，标本来自2000年11月-2006年4月于中国医科大学附属第一医院行非小细胞肺癌切除手术的患者。患者术前均未接受过放化疗。患者平均年龄59岁(20岁-81岁)，其中男性40例，女性30例。非小细胞肺癌标本根据世界卫生组织2004年的分类标准分为腺癌44例，鳞癌26例；其中高分化13例、中分化40例、低分化17例。40例有淋巴结转移，依据国际抗癌联盟(UICC) 1997年修订的非小细胞肺癌P-TNM分期标准：Ⅰ期23例，Ⅱ期22例，Ⅲ期25例。42例非小细胞肺癌及癌旁肺组织标本用于Western blot检测，标本来自于中国医科大学附属第一医院行非小细胞肺癌切除手术的患者。患者术前均未接受过放化疗。其中男性26例，女性16例。非小细胞肺癌标本根据世界卫生组织2004年的分类标准分为腺癌24例，鳞癌18例；其中高、中分化31例、低分化11例，18例有淋巴结转移。

PRDM14兔抗人多克隆抗体PRDM14(Ab28638)购自美国Abcam生物技术公司，SP超敏免疫组织化学试剂盒(KIT-9710)和DAB显色试剂盒(DAB-0031)购自福州迈新生物技术公司，Trizol试剂购自美国Invitrogen公司。

### 1.2 方法

#### 1.2.1 免疫组织化学检测PRDM14的表达

所有切除标本均用10 %甲醛固定，石蜡包埋，制成4 μm厚切片(RM2155，LEICA德国)，经脱蜡、脱苯、水化后，采用链菌素抗生物素蛋白-过氧化物酶法(SP法)检测PRDM14蛋白表达情况。首先高温高压修复抗原，3% H_2_O_2_阻断内源性过氧化物酶的活性，然后使用PRDM14一抗(1:250) 4 ℃湿盒内反应过夜，次日依次滴加生物素标记的二抗和链菌素抗生物素蛋白-过氧化物酶，DAB显色，苏木素复染细胞核；以PBS代替一抗作为阴性对照。

判定标准：每张切片在显微镜(×400)下随机选取10个视野观测，每个视野计数100个肿瘤细胞，计数其中的阳性细胞数，然后取平均值。染色强度分级为：阴性为0，淡黄色为1，棕黄色为2，深棕色为3。以阳性肿瘤细胞数分级：＜5%为0，5%-25%为1，26%-50%为2，＞50%为3。利用染色强度与阳性肿瘤细胞数乘积来评定结果，0为阴性，1为弱阳性，2-4为阳性，6-9为强阳性。

#### 1.2.2 Western blot检测PRDM14的表达

以β-actin为内参，42例新鲜非小细胞肺癌组织和癌旁对照组织标本均快速剪取适量组织，匀浆、破碎、12 000 g离心20 min、取上清液、紫外分光光度计检测蛋白提取物的吸光度，计算蛋白浓度。沸水变性，用10%分离胶、5%浓缩胶的聚丙烯酰胺凝胶电泳分离蛋白后，将蛋白转移到硝酸纤维素膜上，封闭液封闭，TBST冲洗后，分别与一抗、二抗杂交，用ECL成像系统(MF-Chemi BIS 3.2, DNR, Israel)采集图像，测定条带光密度值。

### 1.3 统计学分析

采用SPSS 13.0统计分析软件进行分析，对PRDM14的表达与临床病理特征的关系采用两组资料非参数秩和检验(性别、年龄、组织学类型和淋巴结转移情况)和多组资料非参数秩和检验(TNM分期和分化)。Western blot光密度值分析采用非小细胞肺癌组织和癌旁组织配对t检验。*P*＜0.05为有统计学差异。

## 2 结果

### 2.1 免疫组织化学检测PRDM14的表达

免疫组化方法检测PRDM14在70例肺鳞癌、腺癌和7例癌旁肺组织中的表达情况，结果发现其表达阳性信号定位于胞浆。在70例肺鳞癌、腺癌组织中，有8例为PRDM14阴性表达(11.43%)，9例为PRDM14弱阳性表达(12.86%)，36例PRDM14阳性表达(51.43%)，有17例PRDM14强阳性表达(24.29%)(图 1)。PRDM14在7例癌旁肺组织中弱表达。PRDM14的表达情况与非小细胞肺癌的分化程度和组织学类型有关。PRDM14在高、中分化腺癌、鳞癌中高表达，在低分化腺癌、鳞癌中低表达(*P*=0.046)；PRDM14在腺癌中的表达高于鳞癌(*P*=0.047)；PRDM14的表达水平与患者的性别(*P*=0.820)、年龄(*P*=0.126)、TNM分期(*P*=0.052)和有无淋巴结转移(*P*=0.240)无统计学差异(表 1)。

**1:**
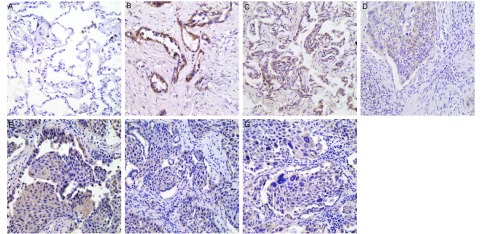
PRDM14在肺腺癌和鳞癌中的表达(SP，×400)。A：PRDM14在癌旁肺组织的表达较弱，细胞浆表达；B：PRDM14在高分化腺癌中高表达，细胞浆表达；C：PRDM14在中分化腺癌中高表达，细胞浆表达；D：PRDM14在低分化腺癌中低表达，细胞浆表达；E：PRDM14在高分化鳞癌中高表达，细胞浆表达；F：PRDM14在中分化鳞癌中高表达，细胞浆表达；G：PRDM14在低分化鳞癌中低表达，细胞浆表达。 The expressions of PRDM14 in lung adencarcinoma and squamous cell carcinoma (SP, ×400). A: The weak positive cytoplasmic expression of PRDM14 in corresponding normal lung; B: The positive cytoplasmic expression of PRDM14 in lung adenocarcinoma; C: The positive cytoplasmic expression of PRDM14 in lung adenocarcinoma; D: The low expression of PRDM14 in lung adenocarcinoma; E: The positive cytoplasmic expression of PRDM14 in lung squamous cell carcinoma; F: The positive cytoplasmic expression of PRDM14 in lung squamous cell carcinoma; G: The low expression of PRDM14 in lung squamous cell carcinoma.

**1:** 免疫组化检测肺癌中PRDM14的表达与临床病理学特征的关系 The expressions of PRDM14 and clinicopathologic characteristics in lung squamous cell carcinoma and adenocarcinoma

Characteristics	*n*	Expression of PRDM14				*Z*	*X*^2^	*P*
		Negative	Weak	Moderate	Strong			
Gender						-0.484		0.629
Male	40	2	5	25	9			
Female	30	6	4	11	8			
Age						-1.531		0.126
＜59 years	28	6	4	11	8			
≥59 years	42	2	5	25	9			
TNM stage							5.900	0.052
Ⅰ	23	2	4	13	4			
Ⅱ	22	0	0	13	7			
Ⅲ-Ⅳ	25	6	5	2	12			
Differentiation							6.147	0.046
Highly differentiated	13	2	2	7	0			
Moderately differentiated	40	6	5	18	11			
Poorly differentiated	17	0	2	3	12			
Histological type						-1.982		0.047
Squamous cell carcinoma	26	5	3	8	8			
Adencarcinoma	44	6	6	20	15			
Lymphnode metastasis						-1.174		0.240
No	30	4	4	13	8			
Yes	40	4	5	15	15			

### 2.2 Western blot检测PRDM14的表达

42例癌旁组织中33例出现特异性目的条带(64 kDa)，42例非小细胞肺癌组织中36例出现特异性染色条带(64 kDa)(图 2)。对癌旁与非小细胞肺癌组织的光密度值分别与β-actin光密度值的比值进行配对t检验，结果显示癌旁组织与肺鳞癌、腺癌中PRDM14的表达水平存在统计学差异。PRDM14在肺鳞癌、腺癌中的表达显著高于癌旁肺组织(*P*＜0.001)。而且PRDM14的表达与非小细胞肺癌的分化程度有关(表 2)，PRDM14在高中分化的非小细胞肺癌组织中的表达高于低分化的非小细胞肺癌组织(*P*=0.017)。而PRDM14的表达与组织学类型(*P*=0.879)和淋巴结转移无统计学差异(*P*=0.455)。

**2:**
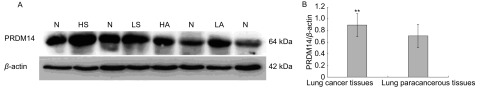
PRDM14蛋白在肺癌组织和癌旁组织中的表达。A：PRDM14蛋白在肺癌组织和癌旁组织表达的Western blot检测结果。N：癌旁组织；HS：高分化鳞癌；LS：低分化鳞癌；HA：高分化腺癌；LA：低分化腺癌。B：癌旁组织中PRDM14的表达量明显低于非小细胞肺癌组织，^**^P＜0.001。 The expressions of PRDM14 in the non-small cell lung cancer tissue and paracancerous tissue. A: The Western blot results showed the expressions of PRDM14 in the non-small cell lung cancer tissue and paracancerous tissue; N: Paracancerous tissue; HS: High differentiated squamous cell carcinoma; LS: Low differentiated squamous cell carcinoma; HA: High differentiated adencarcinoma; LA: Low differentiated adencarcinoma. B: The expression of PRDM14 in paracancerous tissue was lower than that of non-small cell lung cancer tissue, ^**^P < 0.001.

**2:** PRDM14表达的Western blot检测结果与临床病理学特征的关系 The relationship of expressions of PRDM14 in lung sqnamous cell carcinoma and adenocarcinoma tested by Western blot and clinicopathologic characteristics

Characteristics	*n*	Expression of PRDM14	*t*	*P*
Histological type			-1.52	0.879
Squamous cell carcinoma	17	0.93		
Adencarcinoma	25	0.87		
Differentiated			-2.59	0.017
Highly & moderately differentiated	31	0.91		
Poorly differentiated	11	0.86		
Lymph node metastasis			1.70	0.455
No	24	0.93		
Yes	18	0.85		

## 3 讨论

PRDM是一个与人类肿瘤形成有关的转录调节因子家族^[1]^，*PRDM*基因编码的蛋白对维持细胞的完整性和控制细胞的分化、生长及凋亡起着关键作用^[6]^。PRDM家族成员均具有PR-domain和不同数目的重复锌指序列，家族成员主要功能与组氨酸去乙酰化及甲基化有关，在人类癌症中主要起到抑制肿瘤细胞的作用^[7]^。PRDM家族成员与肿瘤的发生有关，PRDM1和PRDM2均编码一个C-MYC转录抑制物，均可促进B淋巴细胞成熟，导致生长停止并显示凋亡前活性，其与大细胞淋巴瘤的发生有关^[8]^。PRDM3在骨髓组织化生中通常是变异的，与多发性骨髓瘤有关^[9]^，而且与造血系统恶性肿瘤的发生有关^[10]^。PRDM4位于肿瘤抑制物区12q，而且在DNA合成中过表达^[2]^。PRDM5在乳腺癌、卵巢癌和肝癌中保持沉默^[1]^。PRDM13也与肿瘤形成有关^[11]^。PRDM16已经被认为与急性髓细胞性白血病相关^[12-14]^。另外，PRDM9与卵巢癌相关^[15]^，PRDM15与胰腺癌也相关^[16]^。据报道^[4]^PRDM14在乳腺癌组织中过表达，但目前PRDM14在其它癌症中的表达情况还不清楚。

本研究中检测了PRDM14在非小细胞肺癌中的表达水平，免疫组化结果显示，在癌旁肺组织中PRDM14弱表达，在肺鳞癌和腺癌中PRDM14较高表达。Noriko等^[4]^研究也发现在正常乳腺组织里几乎没有PRDM14的表达，而在乳腺癌中PRDM14常常过表达；有研究^[17]^表明PRDM14在Evi32原病毒整合的肿瘤中过表达；刘艳艳等^[18]^研究PRDM14的同族因子PRDM1在非小细胞肺癌中表达水平的免疫组化结果中PRDM1也是在鳞状细胞癌中优势表达；与本研究中抑癌家族PRDM成员PRDM14在肿瘤中的异常表达情况是一致的。可能是PR区域家族成员在癌症发生过程中的一种异常的“阴-阳”调节方式的作用，或与组氨酸去乙酰化及甲基化有关^[19]^。

本研究统计分析结果表明PRDM14的表达情况与非小细胞肺癌的分化程度和组织学类型有关。随着分化程度的升高PRDM14的表达水平也升高，推测PRDM14在肺癌发生的早期起作用，促进细胞增殖，在肺癌发展到严重分化不良时，可能由于PR域的CpG岛甲基化使mRNA表达受到抑制，引起了其蛋白表达的下调。另用Western blot检测了PRDM14蛋白在非小细胞肺癌组织中的表达情况，结果显示PRDM14的蛋白表达在肺鳞癌和腺癌中均高于癌旁肺组织，而且与非小细胞癌的分化程度有关，与非小细胞肺癌组织免疫组化检测的结果一致，但与组织学类型无关，这可能是病例样本相对于免疫组化样本例数较少，有待于扩大样本进一步研究。

Noriko等^[4]^研究还发现异常表达的PRDM14促进了肿瘤细胞的生长并降低了肿瘤细胞对化疗药物的敏感性，PRDM14的第二内含子增加了DNA甲基化水平，在乳腺癌细胞中增加的甲基化区域与PRDM14高表达有关，而且其原因与基因扩增有关。刘艳艳等^[18]^对PRDM14的同家族基因*PRDM1*在非小细胞肺癌的表达研究中发现，PRDM1在肺鳞癌中高度表达，肺鳞癌中PRDM1在转录和蛋白水平上均存在异常。本研究中PRDM14在非小细胞肺癌组织中的高表达具体机制是否与甲基化、基因扩增有关以及在转录和蛋白水平上是否存在异常有待进一步研究。
